# Association between Functional Fitness and Health-Related Quality of Life in the Balearic Islands’ Old Adults with Metabolic Syndrome

**DOI:** 10.3390/nu14091798

**Published:** 2022-04-25

**Authors:** Javier Conde-Pipó, Cristina Bouzas, Miguel Mariscal-Arcas, Josep A. Tur

**Affiliations:** 1 Department of Didactics of Musical, Plastic and Corporal, Faculty of Education Sciences, University of Granada, 18071 Granada, Spain; javiercondepipo@gmail.com; 2 Research Group on Community Nutrition and Oxidative Stress, University of the Balearic Islands-IUNICS, 07122 Palma de Mallorca, Spain; cristina.bouzas@uib.es; 3 Health Institute of the Balearic Islands (IDISBA), 07120 Palma de Mallorca, Spain; 4 CIBEROBN (Physiopathology of Obesity and Nutrition CB12/03/30038), Instituto de Salud Carlos III (ISCIII), 28029 Madrid, Spain; 5 Department of Nutrition and Food Science, School of Pharmacy, University of Granada, 18071 Granada, Spain; mariscal@ugr.es

**Keywords:** metabolic syndrome, physical activity, fitness, quality of life, older adults

## Abstract

Research assessing the relationship between functional fitness (FF) and health-related quality of life (HRQoL) is still scarce. The objective of this research is to assess the association between FF and HRQoL in older adults with metabolic syndrome (MetS) from Balearic Islands (Spain). The design is a cross-sectional, descriptive, and comparative study involving 209 participants (42.2% women). The sociodemographic data and medical history of the participants were collected. Physical activity was evaluated using the Spanish version of the Rapid Assessment of Physical Activity Questionnaire. Anthropometrics and blood pressure were measured. Glucose, total cholesterol, high-density lipoprotein cholesterol, and triglyceride plasma levels were measured. A battery of functional fitness tests was applied. HRQoL was measured with the Spanish version of the SF-36 questionnaire. Adherence to the Mediterranean dietary pattern was assessed. In older subjects with MetS, a higher FF score and, within it, endurance, lower body strength, one-leg balance, and agility are positively associated with lower physical function (*p* < 0.001; d = 0.56), better general health (*p* = 0.019; d = 0.35), and better summary physical component of HRQoL (*p* < 0.001; d = 0.57). The FF score and HRQoL physical component are both positively associated with high levels of physical activity (ORadj = 10.3, IC 4.19–28.2, *p* < 0.001; ORadj = 3.25, IC 1.44–7.72, *p* < 0.005). Older adults with MetS should consider practicing physical activity above the general recommendations to improve their functional fitness and health status and quality of life.

## 1. Introduction

Metabolic syndrome (MetS) is a set of interrelated risk factors for cardiovascular diseases (CVD), atherosclerosis, and type II diabetes mellitus [1,2] responsible for both a 2-fold increase in the risk of coronary heart disease and the risk of cerebrovascular disease, as well as a 1.5-fold increase in the risk of all-cause mortality [3]. The main factors that constitute it are hyperglycemia, high blood pressure, high levels of triglycerides, low levels of cholesterol linked to high-density lipoproteins, and abdominal obesity [1]. The average prevalence of MetS is 20% to 30% of the adult population in developed countries and tends to increase [4,5], which is why today it is one of the main challenges in public health [2]. In Spain, the prevalence is 31%, with the highest values corresponding to the Balearic Islands, with 33.50% [6]. The world population is aging rapidly, in such a way that in 2050, it is expected that the percentage of adults over 65 years of age will represent 17% of the world population [7], further aggravating the problem of the high prevalence of MetS, since this population group is especially sensitive to MetS risk factors [4].

The causes responsible for MetS are multifactorial and include family history and lifestyle, and there is scientific evidence on the suitability of implementing strategies based on healthy nutrition, the avoidance of sedentary behaviors, and the regular practice of intense physical activity, moderate or vigorous, to prevent and reduce metabolic decline [1,8,9,10,11,12]. The practice of physical activity in the elderly is also linked to a slowdown in the loss of functional fitness, understanding it as the ability to carry out daily activities independently, safely, and without presenting fatigue [13]. Functional fitness (FF) tends to decline with the natural aging process, losing muscle strength, aerobic capacity, flexibility, balance, and agility, and thus hindering the normal daily functioning of older adults [14], who, from 60 years of age and even being healthy, present a two times greater risk of acquiring a disability and four times greater of presenting a functional limitation [15].

Therefore, in older adults with MetS, low FF could negatively influence not only metabolic health but also health-related quality of life (HRQoL), a multidimensional concept that encompasses both physical and mental functioning. Understanding how FF and HRQoL are related globally, and which of the FF components (strength, endurance, flexibility, balance, and agility) is basic in this relationship, could facilitate the development and implementation of specific interventions that lead to the improvement of HRQoL in older adults with MetS.

Although there are numerous studies that establish a positive relationship between physical activity and HRQoL [16], research assessing the relationship between FF and HRQoL [17] is still scarce. Moreover, these studies have several limitations: first, in terms of sample, either because they assessed populations with lifestyle patterns very different from those of Europe, such as Asia [13], or they were made up mostly of women [18,19] or they were a healthy population or with some pathology other than MetS [20], and second, regarding the chosen variables, since several studies only contemplate some of the physiological abilities of FF in isolation [14,17,21].

According to these limitations and existing findings, the authors of this paper hypothesize that there might be a relationship between quality of life and functional fitness in this population group.

Therefore, the aim of this study was to assess the association between functional fitness and health-related quality of life in older adults with MetS from the Balearic Islands (Spain).

## 2. Methods

### 2.1. Design and Subjects

**Figure 1:**
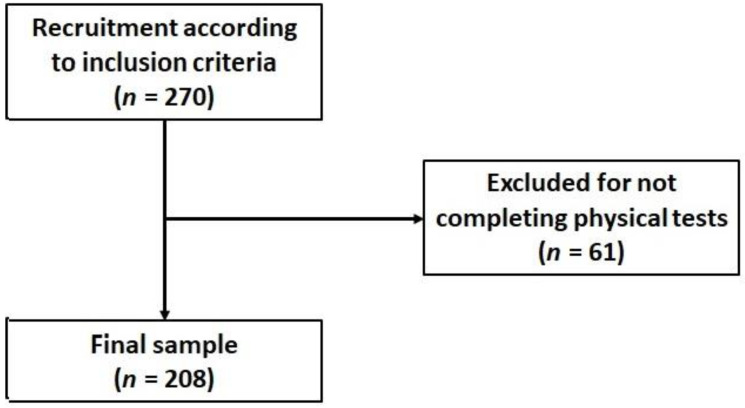
Study flowchart.

The study design was cross-sectional, descriptive, and comparative. The study protocols and procedures were developed in accordance with the standards of the Declaration of Helsinki and approved by the Research Ethics Committee of the Balearic Islands (CEIC-IB2251/14PI and CEIC-IB1295/09PI). Participants were recruited throughout the cardiovascular disease unit of the “Son Llàtzer” University Hospital and the Balearic Islands Primary Health Care Service, where all the tests were carried out. Prior to participating in the study, all of them were informed of the aim of the research and provided their written informed consent. According to the data of the Spanish National Statistics Institute (https://www.ine.es/ accessed on 28 March 2022) about the population of 55- to 75-year-old residents in Balearic Islands, and the prevalence of MetS in this population, it was estimated that a sample size of at least 207 participants would be sufficient under the conditions of α = 0.05 and two-sided confidence interval = 90%. The sample was initially composed of 270 subjects (Figure 1), residents of the Balearic Islands. The inclusion criteria were age (55–75 years) and suffering from MetS, while the exclusion criteria consisted of being institutionalized, suffering from some physical or mental illness that could limit the evaluation of physical fitness or the ability to respond to questionnaires, drug addiction, or chronic alcoholism. Of all those selected, 61 were discarded for not completing the physical evaluation, so the final sample was made up of 209 subjects (42.2% women).

### 2.2. Sociodemographic and Lifestyle Determinants

The sociodemographic data, such as sex, age, marital status (married, divorced, single, or widow), employment status (retired or active), level of education (primary, secondary, or university), and medical history—previous illnesses, drug consumption, and smoking (nonsmoker; ex-smoker: more than 1 year with no smoking; or smoker: ≥1 cigarette/day)—of the participants were collected.

### 2.3. Physical Activity

Physical activity was evaluated using the Spanish version of the Rapid Assessment of Physical Activity Questionnaire (RAPA-Q) [22], a validated questionnaire that has seven items, is easy to use, has demonstrated reliability and sensitivity, is administered by trained interviewers in individual sessions, and is specifically designed for its use in adults over 50 years of age, since in addition to assessing the intensity and frequency of weekly physical activity, it includes two items that separately assess strength and flexibility, both important for this group of the population due to their relevance in the prevention of falls and loss of autonomy [23,24]. This questionnaire can be answered affirmatively (yes) or negatively (not), and allows an easy identification of the level of PA based on whether the WHO recommendations of minimum practice are achieved to obtain benefits on cardiovascular health [23]. Thus, the level was classified as low (when activities between light and moderate were practiced but not every week), moderate (when these activities were practiced less than 150 min per week or 75 min in the case of vigorous activities), or high (when these time thresholds were exceeded) [25].

### 2.4. Anthropometrics, Blood Pressure, and Blood Analytical

Anthropometric variables were taken by trained personnel. Height was measured in centimeters using a wall-mounted stadiometer (Seca 214, SECA Deutschland, Hamburg, Germany), and weight in kilograms with a high-precision scale (Tanita BC-418, Tanita, Tokyo, Japan). All participants were weighed barefoot and wearing light clothing, subtracting 0.6 kg from the total for clothing [1]. Body mass index (BMI) was calculated by dividing weight in kilograms by the square of height in meters (kg/m^2^). Waist circumference (WC) was measured with a tape measure at the mid-distance between the iliac crest and the last rib [26].

Blood pressure was measured with a validated semiautomatic oscillometer (Omron HEM-705CP, Hoofddorp, The Netherlands), leaving 5 min between measurements and in a sitting position. All anthropometric measurements were taken in duplicate using the mean of both for subsequent analysis.

Blood samples were collected in the antecubital vein in the morning and after an 8 h fast. Biochemical analyses (glucose, total cholesterol, high-density lipoprotein cholesterol (HDL-c), and triglyceride (TG) plasma concentration) were performed in local laboratories using Abbott Architect c16000 employing commercial kits (Abbott Diagnostics, Lake Bluff, IL, USA).

### 2.5. Metabolic Syndrome Assessment

The diagnosis of metabolic syndrome (MetS) was confirmed according to the updated and harmonized definition of the International Diabetes Federation, the American Heart Association, and the National Heart, Lung, and Blood Institute, according to which it is necessary to present three of the five risk factors (WC > 102 cm in men and ≥88 in women; TG ≥ 150 mg/dL (1.7 mmol/L); HDL-c < 40 mg/dL (1.0 mmol/L) in men and <50 mg/dL (1.3 mmol/L) in women; systolic blood pressure ≥ 130 and diastolic ≥ 85 mmHg); fasting glucose ≥ 100 mg/dL) [1,2].

### 2.6. Functional Fitness Test Battery

The battery of functional fitness tests is a validated, reliable, simple-to-administer, and noninjurious tool developed to evaluate in older adults the physiological abilities involved in the execution of the main daily tasks in a safe, autonomous, and fatigue-free way [15,27]. Endurance was evaluated using the 2-min step test [15,28], lower body strength using the 30” chair stand test [15,25], upper body strength using the standing and sitting handgrip test [28,29], agility and dynamic balance using the 8-foot time up and go test [30,31], flexibility of the upper body using the chair sit-and-reach test and that of the lower body using the back scratch test [32], and static balance using the 60 s one leg balance test [32,33]. All the tests were carried out on the same day and in the same order, always being supervised by trained personnel. The absence of cardiovascular contraindications for the practice of exercise was previously confirmed in all participants through the Physical Activity Readiness Questionnaire [34]. To assess functional health globally, a functional fitness score (FFS) was constructed. To do this, each participant was assigned one point for each functional test whose result was equal to or greater than the 50th percentile of his or her gender, obtaining the FFS value after adding all the points, having a range between 0 and 8. Subsequently, the variable was categorized into two levels, “high” and “low” according to the score equal to or lower than the 50th percentile of the corresponding gender or higher than that.

### 2.7. Health-Related Quality of Life

Health-related quality of life was measured with the Spanish version of the SF-36 [17,35] questionnaire, which has proven validity and high reliability and is widely used in the elderly population for measuring subjective health and abilities or limitations to performing tasks daily [25,29,36]. It is composed of eight domains: physical role (10 items), physical function (4 items), body pain (2 items), general health (5 items), social function (2 items), emotional health (3 items), mental health (5 items), and vitality (4 items). Each domain is scored on a scale of 0 to 100, with the highest score corresponding to the best health status. It also provides two summary components, the physical component (COMP-F) and the mental component (COMP-M), which are calculated with specific weights for the Spanish population [37]. To establish the cut-off points that allowed both summary components to be classified into two levels, the medians of the tables of Spanish population norms according to age groups and gender were used [37].

### 2.8. Adherence to the Mediterranean Dietary Pattern

To assess adherence to the Mediterranean dietary pattern (MDP), an adjusted energy intake value was obtained for everyone according to the daily consumption of legumes, cereals (including bread and potatoes), fruits, vegetables, meat (including meat products), and milk (including dairy products). Information on the consumption of all these foods was obtained from the 137-item food frequency questionnaire (FFQ), previously validated in Spain [38]. For each item, a typical portion size was included, and consumption frequencies were recorded in nine categories ranging from “never or hardly ever” to “≥6 times/day”. Energy and nutrient intakes were calculated by multiplying the frequency by the nutrient composition of the portion size specified for each food using a proprietary computer program based on the information available in the Spanish food composition tables [39]. The monounsaturated fatty acid (MUFA)/saturated fatty acid (SFA) ratio was also calculated. To score “moderate alcohol consumption”, a transformation centered on the level of consumption of 30 g/day for men and 20 g/day for women was used. All these values were standardized as a Z value [40,41]. A total Mediterranean diet score (MDS) was calculated by adding all the Z scores obtained for the favorable or more Mediterranean dietary components (legumes, cereals and roots, fruits, vegetables, fish, moderate alcohol, MUFA/SFA ratio) and subtracting the Z value obtained from the consumption of meat and milk, according to the formula previously described in the scientific literature [1,40,42]. The MDS was converted to percent adherence using the range of values for the sample. The participant with the highest adherence value in the sample was assigned 100% adherence, and the participant with the lowest value 0%. Low adherence to the Mediterranean diet (MD) was defined as a percentage of adherence below the 25th percentile, medium adherence was defined as a percentage of adherence between the 25th and 75th percentiles, and high adherence was defined as a percentage of adherence above the 75th percentile.

### 2.9. Statistics

Statistical analysis was performed with the R statistical computing software (R Core Team, Vienna, Austria). The normality of the variables was analyzed using the Kolmogorov–Smirnov test with the Lilliefors correction, and the homoscedasticity with the Levene test. For the basic descriptions, frequencies, means, and standard deviations were used. For comparisons between groups of continuous variables, the nonparametric tests for independent variables, Mann–Whitney U and Kruskal–Wallis, were used, as appropriate, and the Cohen d and eta-square indices were used to calculate the effect size. For comparisons between groups of categorical variables, Pearson’s chi-squared test was used. In the case of bivariate correlations, Spearman’s rho correlation coefficient was used. Logistic regression models were applied to assess the association between the frequency of high levels of FF, COMP-F, and COMP-M with various health, lifestyle, and sociodemographic factors. For each of these models, the joint effect of all the variables that make up the model was analyzed (or adjusted). The results are presented as crude odds ratios (OR_cr_) and adjusted odds ratios (OR_adj_) with 95% confidence intervals. The internal reliability of the instruments was evaluated using Cronbach’s alpha coefficient. All reported *p*-values were based on the two-tailed test, and the level of statistical significance for all tests was set at 95%.

## 3. Results

The initial characteristics of the sample analyzed by gender, as well as the scores obtained in FFS, COMP-F, and COMP-M, are shown in Table 1. The final sample consisted of 209 subjects, of which 57.8% were men (*n* = 121) and 42.20% were women (*n* = 88). Differences were found in gender, age, height, weight, waist circumference, percentage of obesity, adherence to the Mediterranean diet, smoking, marital status, educational level, COMP-F, and COMP-M. No other differences were found.

Table 2 shows the bivariate correlations between the physical abilities measured by the functional fitness test battery, Mediterranean dietary adherence (MDA), and dimensions of SF-36. The SF-36 subdomain that presented the highest correlations (r ≥ 0.30) was physical role, highlighting the moderate correlation obtained with lower body flexibility (r = 0.51), followed by endurance (r = 0.43), one-leg balance (r = 0.41), standing handgrip (r = 0.40), sitting handgrip (r = 0.39), and lower body strength (r = 0.36). The correlation with upper body flexibility and agility was not significant. Both social function and emotional health did not correlate significantly with any of the physical abilities. No significant correlation was found between MDA and SF-36 or physical abilities.

Table 3 shows the crude and adjusted results of the logistic regression models that evaluate the association between health (model 1), lifestyle (model 2), and sociodemographic (model 3) factors with the functional fitness, physical, and mental components of health-related quality of life associated. Having a high level of FF score was positively associated with high levels of physical activity level (OR_adj_ = 10.3, 95% CI 4.19–28.2, *p* < 0.001) and not being retired (OR_adj_ = 2.17, 95% CI 1.39–3.43, *p* < 0.001) and inversely associated with suffering from five metabolic pathologies (OR_adj_ = 0.19, 95% CI 0.05–0.56, *p* = 0.005), having type II or III obesity (OR_adj_ = 0.18, 95% CI 0.07–0.44, *p* < 0.001), nonsmoking (OR_adj_ = 0.23, 95% CI 0.08−0.63, *p* = 0.004), and having only completed primary school (OR_adj_ = 0.41, 95% CI 0.45–2.41, *p* = 0.034). Presenting values of the physical health component above the population mean was positively associated with a high FF score (OR_adj_ = 1.89, 95% CI 1.02–3.53, *p* = 0.044), with high levels of PA (OR_adj_ = 3.25, 95% CI 1.48–7.50, *p* = 0.003) and mean PA levels (OR_adj_ = 2.21, 95% CI 1.01–4.51, *p* = 0.046). Regarding the mental health component, presenting values above the population mean were inversely associated with a high FF score (OR_adj_ = 0.49, 95% CI 0.26–0.91, *p* = 0.025), not being retired (OR_adj_ = 0.62, 95% CI 0.40–0.95, *p* = 0.030), and being widowed (OR_adj_ = 0.23, 95% CI 0.06–0.67, *p* = 0.010).

The association between the number of metabolic pathologies, physical abilities, and FFS, for the entire sample and differentiated by gender, is shown in Table 4. In the group with five metabolic diseases, they were significantly lower, both for the entire sample and for men, FFS, endurance, lower body strength, and agility. For the entire sample and only women, it was the lower body flexibility. The only variable with significantly smaller differences in men, women, and the entire sample was one-leg balance.

The association between the levels of the FFS (high/low) and the dimensions of SF-36 is shown in Table 5. For the high FFS group, and for the entire population, the highest values and strongest association of function were obtained in physical function (*p* < 0.001; d = 0.56), COMP-F (*p* < 0.001; d = 0.57), and COMP-M (*p* = 0.013; d = 0.57), followed by general health (*p* = 0.019; d = 0.35). The physical role, body pain, emotional health, vitality, and mental health subdomains did not show a significant association.

Finally, Table 6 shows the association between the FFS and the physical abilities that make it up, and the physical and mental components categorized into two groups, the one with a score less than or equal to the population median and the one with a score higher than the median. Considering the sample globally, the group with the best physical health (>Mep) was the one that obtained the highest score in all the tests, giving significant differences in both the FFS (*p* = 0.003; d = 0.42; CI = 0.15−0.70) and endurance (*p* < 0.001; d = 0.52; CI = 0.23–0.80), lower body strength (*p* < 0.001; d = 0.47; 0.20–0.75), one-leg balance (*p* = 0.011; *p* = 0.32; CI = 0.04–0.59), and agility (*p* = 0.052; d = 0.32; CI = 0.05–0.59).

## 4. Discussion

The main finding of this study was that in older subjects with MetS, a high level of functional fitness is positively associated with the physical component of health-related quality of life (HRQoL), with the differences being greater in those people who suffer from five of the pathologies of which this syndrome is composed.

Among the components of the FF that influence this relationship with the HRQoL physical component, the main one was endurance, followed by lower body strength, and to a lesser extent, balance and agility. These results are consistent with previous studies that analyzed this relationship [13,17], so they should be included in the exercise recommendations in this phase of life.

Maintaining an optimal level of functional fitness plays an important role at any time in life, but it is especially important in the elderly since it allows them to maintain mobility and autonomy, determining the performance of day-to-day tasks, participating in social gatherings, and ultimately, maintaining the quality of life and well-being [18].The loss of the FF with age is inevitable, but it can be stopped with the practice of physical activity [14]. However, despite the weekly recommendations of the WHO [43] regarding the practice of PA, in the current results, only regular practice above 150 min per week at moderate intensity or above 75 min in case of vigorous activities is associated with a higher FF. It indicates that these recommendations could be insufficient to improve the FF in older adults with MetS, requiring greater physical intensity stimuli. It should be considered as a guide when recommending and planning physical activities aimed at this population. Not only the lack of deliberate physical activity is negatively associated with the FF. Being retired, a situation typical of the population over 65 years of age, despite implying having more free time, is also a stressful situation that can lead to depressive, sedentary, and unhealthy behaviors due to loss of identity [44], which lead to a decrease in notable increase in daily activity and thus in the FF, as shown by current results, and in line with those of other previous similar studies [45]. However, regarding mental health, the association found in this study is positive, probably due to the loss of work stress and the greater availability of leisure time available to retired people [44].

The current study also shows that obesity is a factor that is negatively associated with the FF and COMP-F. This could be a consequence of the inverse association between obesity and the practice of physical activity [46]; however, excess weight and adipose tissue limits movement ability, decreases cardiovascular capacity, favors a loss of muscle mass, modifies the posture and biomechanics of the body, and increases instability and pain in the joints and back due to wear of the cartilage in the knees and the compression of the intervertebral discs [47,48,49].

Similar to previous research [13], current findings showed that physical function was the HRQoL subdomain with the greatest association with high levels of FF and somewhat weaker general health; on the contrary, no association was found with other subdomains, except for emotional health (degree to which emotional problems interfere with work or other daily activities), which was inversely associated, an issue that should be further investigated. Similarly, other authors [29] have also found a positive association between improvement in physical fitness after completing interval training programs and high intensity with respect to the limitation of activities and general health subdomains.

### Strengths and Limitations of the Study

The current study shows, as main strength, that a high level of functional fitness in older subjects with MetS is positively associated with the physical component of health-related quality of life (HRQoL). This study has several limitations. First, the cross-sectional study limits the ability to establish a cause–effect relationship between functional fitness and health-related quality of life in the presence of MetS. Second, the data obtained regarding the practice of physical activity and eating habits were reported by the participants, lacking objective data, so their interpretation must be performed with caution. Third, given that the sample studied were residents of the Balearic Islands, generalization to other populations may be limited.

## 5. Conclusions

In conclusion, the current study shows that in older subjects with metabolic syndrome (MetS), a higher functional fitness score and, within it, aerobic endurance, lower body strength, one-leg balance, and agility are positively associated with lower physical role, better general health, and better summary physical component of health-related quality of life (HRQoL). In turn, both the FFS and the physical component are positively associated with high levels of physical activity. Accordingly, older adults with MetS should consider practicing physical activity above the general recommendations of the WHO to improve their functional fitness, and with it their health status and quality of life.

## Figures and Tables

**Table 1:** Characteristics of the study sample by sex.

	Men	Women	*p*
Sample (*n*; %)	121 (57.8)	88 (42.2)	0.022 *
Age (years; mean and SD)	63.3 (5.6)	65.6 (4.2)	0.001 *
Height (cm; mean and SD)	169.3 (6.1)	155.1 (5.7)	0.001 *
Weight (kg; mean and SD)	93.3 (13.3)	79.5 (10.7)	0.001 *
BMI (kg/m^2^; mean and SD)	32.4 (3.5)	33.03 (3.8)	0.261 *
Waist circumference (cm; mean and SD)	113.9 (9.5)	106.6 (9.4)	0.001 *
Obesity (*n*; %)	121 (57.8)	88 (42.2)	0.022 ^#^
Metabolic pathologies (*n*; %)			
3	57 (47.1)	48 (54.5)	0.440 ^#^
4	42 (34.7)	29 (33.0)	
5	22 (18.2)	11 (12.5)	
Physical activity (*n*; %)			
Low	77 (63.6)	57 (64.8)	0.972 ^#^
Medium	25 (20.7)	17 (19.3)	
High	19 (15.7)	14 (15.9)	
Adherence to MedDiet (*n*; %)			
Low (<p25)	54 (45.4)	19 (21.6)	0.001 ^#^
Medium (p25–p75)	45 (37.8)	43 (48.9)	
High (>p75)	20 (16.8)	26 (29.5)	
Smoking (*n*; %)			
Smoker	19 (15.7)	10 (11.5)	0.001 ^#^
Ex-smoker	72 (59.5)	30 (34.5)	
Nonsmoker	30 (24.8)	47 (54.0)	
Civil status (*n*; %)			
Married	98 (81.0)	63 (71.6)	0.001 ^#^
Divorced	15 (12.4)	1 (1.1)	
Single	4 (3.3)	9 (10.2)	
Widow	4 (3.3)	15 (17.0)	
Educational level (*n*; %)			
Primary	45 (37.2)	55 (62.5)	0.001 ^#^
Secondary	44 (36.4)	20 (22.7)	
University	32 (26.4)	13 (14.8)	
Employment status (*n*; %)			
Retired	63 (52.5)	54 (61.4)	0.261 ^#^
Active	57 (47.5)	34 (38.6)	
Physical and health tests (mean; SD)			
Functional fitness score	3.89 (2.2)	3.78 (2.3)	0.726 *
Physical health component	48.14 (7.5)	44.37 (9.4)	0.008 *
Mental health component	52.05 (10.4)	46.79 (12.2)	0.001 *

Abbreviations: BMI: body mass index; MedDiet: Mediterranean diet. Differences between women and men by * Mann–Whitney nonparametric test and by ^#^ Pearson’s chi-squared test.

**Table 2:** Correlation between physiological abilities, Mediterranean dietary adherence, and SF-36 components.

Variable	Lower-Body Strength	Endurance	One-Leg Balance	Standing Handgrip	Sitting Handgrip	Agility	Upper-Body Flexibility	Lower-Body Flexibility	Physical Role	Physical Function	Body Pain	General Health	Social Functioning	Emotional Health	Mental Health	Vitality
Endurance	0.58 **															
	(0.48, 0.67)															
One-leg balance	0.30 **	0.34 **														
	(0.17, 0.42)	(0.21, 0.46)														
Standing handgrip	0.28 **	0.33 **	0.35 **													
	(0.15, 0.40)	(0.20, 0.45)	(0.22, 0.46)													
Sitting handgrip	0.28 **	0.32 **	0.34 **	0.97 **												
	(0.15, 0.40)	(0.19, 0.44)	(0.21, 0.46)	(0.96, 0.98)												
Agility	0.16 *	0.13	0.11	0.02	0.01											
	(0.02, 0.29)	(−0.01, 0.27)	(−0.03, 0.24)	(−0.12, 0.16)	(−0.13, 0.14)											
Upper-body flexibility	0.13	0.14	0.09	−0.08	−0.08	0.24 **										
	(−0.01, 0.26)	(−0.00, 0.27)	(−0.04, 0.23)	(−0.21, 0.06)	(−0.21, 0.06)	(0.11, 0.37)										
Lower-body flexibility	−0.51 **	−0.48 **	−0.53 **	−0.39 **	−0.37 **	−0.12	−0.17 *									
	(−0.60, −0.40)	(−0.58, −0.36)	(−0.62, −0.43)	(−0.50, −0.26)	(−0.48, −0.24)	(−0.25, 0.01)	(−0.30, −0.04)									
Physical role	0.17 *	0.27 **	0.12	0.11	0.11	−0.06	0.03	−0.21 **								
	(0.03, 0.30)	(0.13, 0.40)	(−0.02, 0.25)	(−0.02, 0.25)	(−0.03, 0.24)	(−0.20, 0.07)	(−0.10, 0.17)	(−0.33, −0.07)								
Physical function	0.36 **	0.43 **	0.41 **	0.40 **	0.39 **	0.12	0.01	−0.51 **	0.40 **							
	(0.23, 0.47)	(0.31, 0.54)	(0.29, 0.52)	(0.27, 0.50)	(0.27, 0.50)	(−0.01, 0.26)	(−0.12, 0.15)	(−0.61, −0.40)	(0.28, 0.51)							
Body pain	0.19 **	0.23 **	0.15 *	0.25 **	0.23 **	0.00	−0.07	−0.19 **	0.44 **	0.55 **						
	(0.05, 0.31)	(0.09, 0.36)	(0.01, 0.28)	(0.12, 0.37)	(0.10, 0.36)	(−0.13, 0.14)	(−0.21, 0.06)	(−0.32, −0.05)	(0.32, 0.54)	(0.45, 0.64)						
General health	0.10	0.17 *	0.26 **	0.18 **	0.17 *	0.12	0.14 *	−0.23 **	0.22 **	0.43 **	0.28 **					
	(−0.03, 0.24)	(0.03, 0.30)	(0.13, 0.39)	(0.05, 0.31)	(0.03, 0.30)	(−0.01, 0.25)	(0.01, 0.27)	(−0.36, −0.10)	(0.08, 0.34)	(0.32, 0.54)	(0.14, 0.40)					
Social functioning	−0.01	0.12	0.09	0.14 *	0.14	−0.07	0.01	−0.07	0.39 **	0.24 **	0.15 *	0.16 *				
	(−0.14, 0.13)	(−0.02, 0.26)	(−0.05, 0.22)	(0.01, 0.27)	(−0.00, 0.27)	(−0.21, 0.07)	(−0.13, 0.14)	(−0.20, 0.07)	(0.26, 0.50)	(0.10, 0.36)	(0.01, 0.28)	(0.02, 0.29)				
Emotional role	−0.06	0.02	0.03	0.06	0.07	−0.13	−0.04	−0.09	0.32 **	0.21 **	0.18 **	0.17*	0.59 **			
	(−0.20, 0.07)	(−0.12, 0.16)	(−0.10, 0.17)	(−0.07, 0.20)	(−0.07, 0.20)	(−0.26, 0.01)	(−0.17, 0.10)	(−0.22, 0.04)	(0.20, 0.44)	(0.08, 0.34)	(0.05, 0.31)	(0.03, 0.29)	(0.49, 0.67)			
Mental health	0.10	0.18 *	0.07	0.23 **	0.23 **	0.02	0.00	−0.18 **	0.36 **	0.34 **	0.31 **	0.34 **	0.38 **	0.51 **		
	(−0.04, 0.23)	(0.04, 0.31)	(−0.07, 0.20)	(0.09, 0.35)	(0.09, 0.35)	(−0.11, 0.16)	(−0.13, 0.14)	(−0.31, −0.04)	(0.24, 0.48)	(0.22, 0.46)	(0.18, 0.42)	(0.21, 0.45)	(0.26, 0.49)	(0.40, 0.60)		
Vitality	0.16 * (0.03, 0.29)	0.23 ** (0.09, 0.36)	0.16 * (0.02, 0.29)	0.19 ** (0.06, 0.32)	0.17 * (0.04, 0.30)	0.19 ** (0.06, 0.32)	0.02 (−0.12, 0.16)	−0.23 ** (−0.36, −0.10)	0.38 ** (0.25, 0.49)	0.52 ** (0.41, 0.61)	0.43 ** (0.31, 0.54)	0.43 ** (0.31, 0.53)	0.35 ** (0.22, 0.46)	0.33 ** (0.20, 0.44)	0.58 ** (0.49, 0.67)	
MDA	−0.07 (−0.20, 0.07)	−0.06 (−0.20, 0.08)	0.01 (−0.12, 0.15)	−0.23 (0.35, −0.09)	−0.22 (−0.35, 0.09)	0.26 * (0.12, 0.38)	0.05 (−0.09, 0.19)	0.08 (−0.05, 0.22)	0.08 (−0.05, 0.22)	−0.07 (−0.21, 0.06)	0.00 (−0.13, 0.14)	0.03 −0.11, 0.16)	0.03 (−0.10, 0.17)	0.09 (−0.04, 0.23)	0.03 (−0.11, 0.16)	0.08 (−0.06, 0.21)

* *p* < 0.05; ** *p* < 0.01; by Spearman’s rho correlation test. MDP: Mediterranean dietary adherence.

**Table 3:** Association between health, lifestyle, and sociodemographic factors vs. functional fitness, physical, and mental components.

	Functional Fitness						Physical Component > MeP						Mental Component > MeP					
	Crude Values			Adjusted Values			Crude Values			Adjusted Values			Crude Values			Adjusted Values		
	OR	95% CI	*p*	OR	95% CI	*p*	OR	95% CI	*p*	OR	95% CI	*p*	OR	95% CI	*p*	OR	95% CI	*p*
**Model 1: health factors**																		
Functional fitness score																		
High vs. low	-	-	-	-	-	-	2.14	1.15–4.00	0.012	1.89	1.02–3.53	0.044	0.50	0.27–0.89	0.019	0.49	0.26–0.91	0.025
MetS criteria																		
4 vs. 3	0.85	0.45–1.59	0.623	0.89	0.46–1.72	0.737	1.09	0.59–2.00	0.777	1.16	0.61–2.18	0.651	0.93	0.50–1.71	0.822	0.95	0.51–2.70	0.891
5 vs. 4	0.19	0.05–0.54	0.004	0.19	0.05–0.56	0.005	0.67	0.29–1.50	0.346	0.88	0.35–2.14	0.794	1.25	0.56–2.82	0.588	1.10	0.46–2.70	0.833
Body mass index																		
Obesity I vs. preobesity	0.65	0.33–1.29	0.223	0.61	0.30–1.24	0.179	0.65	0.33, 1.27	0.203	0.70	0.35–1.40	0.318	1.25	0.637–2.44	0.520	1.20	0.59–2.41	0.609
Obesity II or III vs. preobesity	0.19	0.07–0.44	0.001	0.18	0.07–0.44	0.001	0.44	0.20, 0.935	0.034	0.56	0.25–1.26	0.162	1.18	0.561–2.48	0.664	0.92	0.41–2.06	0.856
**Model 2: lifestyle factors**																		
Physical activity level																		
High vs. low	8.09	3.45–20.8	0.001	10.3	4.19–28.2	0.001	3.25	1.48–7.50	0.003	3.25	1.44–7.72	0.005	0.74	0.34–1.59	0.440	0.71	0.32–1.57	0.404
Medium vs. low	1.94	0.93–4.02	0.074	1.83	0.83–3.97	0.126	1.63	0.80–7.50	0.171	2.21	1.01–4.51	0.046	1.16	0.57–2.37	0.685	1.13	0.54–2.40	0.736
Adherence to MedDiet																		
Medium vs. low	1.40	0.72–2.76	0.320	1.93	0.75–4.32	0.090	0.68	0.36–1.29	0.246	0.77	0.39–1.50	0.446	1.57	0.84–2.95	0.159	1.46	0.77–2.78	0.242
High vs. low	1.54	0.71–3.33	0.271	1.80	0.75–4.32	0.186	1.63	0.77–3.47	0.199	1.60	0.73–3.54	0.237	1.69	0.80–3.61	0.170	1.78	0.83–3.88	0.143
Smoking																		
Ex-smoker vs. smoker	0.66	0.28–1.57	0.354	0.41	0.15–1.08	0.073	0.70	0.30–1.63	0.404	0.58	0.24–1.42	0.233	1.31	0.564–3.02	0.523	1.37	0.57–3.25	0.467
Nonsmoker vs. smoker	0.39	0.15–0.96	0.042	0.23	0.08–0.63	0.004	1.56	0.66–3.72	0.313	1.38	0.56–3.42	0.481	0.75	0.315–1.77	0.514	0.77	0.31–1.86	0.561
**Model 3: sociodemographic factors**																		
Sex																		
Women vs. men	1.09	0.60–1.94	0.778	1.47	0.74–2.96	0270	1.31	0.75–2.28	0.355	1.32	0.71–2.46	0.378	0.86	0.497–1.50	0.604	0.89	0.45–1.69	0.722
Employment level																		
Active vs. retired	2.22	1.47–3.41	0.001	2.17	1.39–3.43	0.001	1.02	0.68–1.50	0.930	1.02	0.67–1.55	0913	0.592	0.39–0.87	0.009	0.62	0.40–0.95	0.030
Civil status																		
Divorced	0.91	0.26–2.77	0.871	0.84	0.23–2.76	0.787	0.52	0.15–1.50	0.246	0.57	0.17–1.71	0.339	0.39	0.12–1.12	0.086	0.42	0.13–1.26	0.129
Single	1.91	0.60–6.20	0.264	1.62	0.23–2.76	0.451	0.51	0.13–1.63	0.278	0.43	0.11–1.45	0.197	1.06	0.33–3.63	0.927	1.34	0.40–4.88	0.633
Widow	1.15	0.39–3.15	0.792	0.90	0.45–5.83	0.850	0.48	0.48–3.37	0.618	1.17	0.43–3.20	0.759	0.23	0.07–0.65	0.008	0.23	0.06–0.67	0.010
Educational level																		
Primary vs. university	0.33	0.15–0.70	0.004	0.41	0.18–0.93	0.034	0.89	0.43–1.81	0.747	0.78	0.36–1.69	0.538	1.59	0.78–3.26	0.198	1.37	0.62–2.99	0.429
Secondary vs. university	0.72	0.33–1.55	0.404	1.04	0.45–2.41	0.925	0.71	0.33–1.54	0.393	0.67	0.29–1.50	0.331	0.80	0.96–4.58	0.063	1.89	0.82–4.38	0.132

Abbreviations: CI: confidence interval; MedDiet: Mediterranean diet; MedP: Spanish population median.

**Table 4:** Association between number of pathologies and physical abilities measured by functional fitness battery tests.

Physical Abilities	3 Mean (SD)	4 Mean (SD)	5 Mean (SD)	*p*	Size Effect	
					eta^2^	95% CI
Functional fitness score						
All	4.13 (2.31)	3.90 (2.08)	2.58 (1.87)	0.002	0.05	(0.01–0.10)
Men	4.18 (1.91)	3.74 (1.77)	2.91 (1.44)	0.032	0.06	(0.01–0.14)
Women	3.77 (1.77)	3.93 (1.65)	3.09 (1.76)	0.322	0.00	(0.00–0.06)
Endurance						
All	13.16 (3.66)	13.15 (3.45)	11.27 (2.76)	0.027	0.04	(0.00–0.09)
Men	14.11 (4.07)	13.55 (3.86)	11.18 (2.59)	0.032	0.06	(0.01–0.15)
Women	12.04 (2.77)	12.59 (2.69)	11.45 (3.21)	0.322	0.00	(0.00–0.02)
Lower body strength						
All	69.40 (18.68)	68.48 (21.77)	58.90 (15.81)	0.027	0.04	(0.00–0.08)
Men	74.59 (18.81)	73.24 (22.19)	59.81 (16.24)	0.006	0.06	(0.01–0.14)
Women	62.65 (16.38)	61.78 (19.64)	56.78 (15.47)	0.554	0.00	(0.00–0.07)
Sitting handgrip						
All	29.00 (11.57)	30.15 (10.83)	30.15 (9.88)	0.780	0.00	(0.00–0.02)
Men	37.77 (7.46)	37.44 (6.22)	34.60 (9.13)	0.143	0.02	(0.00–0.08)
Women	18.60 (5.13)	19.61 (6.44)	21.25 (2.63)	0.267	0.03	(0.00–0.10)
Standing handgrip						
All	28.50 (11.76)	30.32 (10.40)	30.52 (10.02)	0.432	0.00	(0.00–0.03)
Men	36.95 (8.71)	37.36 (5.93)	34.83 (9.49)	0.192	0.00	(0.00–0.05)
Women	18.46 (5.09)	20.13 (6.10)	21.90 (3.07)	0.087	0.05	(0.00–0.14)
One-leg balance						
All	32.22 (20.51)	31.53 (19.68)	21.01 (17.81)	0.008	0.04	(0.00–0.09)
Men	36.27 (20.58)	36.37 (18.75)	25.17 (18.40)	0.037	0.03	(0.00–0.10)
Women	27.41 (19.57)	24.51 (19.17)	12.70 (13.80)	0.026	0.05	(0.00–0.14)
Agility						
All	6.01 (1.61)	6.14 (1.38)	6.70 (1.49)	0.018	0.02	(0.00–0.06)
Men	5.61 (1.41)	5.82 (1.28)	6.39 (1.45)	0.044	0.04	(0.00–0.11)
Women	6.49 (1.70)	6.60 (1.41)	7.33 (1.43)	0.133	0.02	(0.00–0.10)
Upper body flexibility						
All	−12.13 (11.98)	−12.53 (13.38)	−18.95 (10.85)	0.005	0.04	(0.00–0.08)
Men	−13.77 (13.60)	−14.68 (13.50)	−18.82 (12.86)	0.250	0.02	(0.00–0.07)
Women	−10–20 (9.49)	−9.41 (12.77)	−19.20 (5.37)	0.004	0.04	(0.00–0.13)
Lower body flexibility						
All	−2.56 (9.29)	−2.08 (9.67)	−3,42 (9.10)	0.721	0.00	(0.00–0.03)
Men	−3.54 (9.88)	−3.81 (10.08)	−2.11 (8.84)	0.847	0.00	(0.00–0.03)
Women	−1.4 (8.50)	0.42 (8.59)	−6.02 (9.46)	0.157	0.00	(0.00–0.02)

Abbreviations: CI: confidence interval; SD: standard deviation.

**Table 5:** Association between functional fitness score and SF-36 components.

SF-36 Components	Low FFS (≤p50) Mean (SD)	High FFS (>p50) Mean (SD)	*p*	Size Effect	
				d	95% CI
Physical role					
All	77.12 (36.04)	84.09 (32.67)	0.144	0.20	(−0.09–0.49)
Men	84.21 (32.62)	83.52 (33.65)	0.947	0.02	(−0.35–0.39)
Women	67.41 (38.11)	82.03 (33.14)	0.057	0.40	(0.04–0.84)
Physical function					
All	75.55 (18.35)	84.94 (14.34)	0.001	0.56	(0.26–0.85)
Men	80.46 (16.15)	89.43 (9.29)	0.001	0.64	(0.26–1.02)
Women	67.50 (20.85)	74.69 (16.85)	0.009	0.37	(0.07–0.81)
Body pain					
All	63.39 (27.98)	66.95 (25.62)	0.367	−0.13	(−0.42–0.16)
Men	68.07 (26.53)	73.20 (21.58)	0.356	−0.21	(−0.58–0.17)
Women	53.95 (27.85)	62.41 (28.46)	0.101	−0.30	(−0.74–0.14)
General health					
All	59.24 (21.37)	66.47 (19.04)	0.019	0.35	(0.06–0.64)
Men	60.50 (19.22)	69.34 (15.62)	0.025	0.49	(0.11–0.87)
Women	55.61 (24.49)	61.16 (22.34)	0.293	−0.23	(−0.67–0.20)
Social function					
All	91.53 (19.26)	88.47 (20.25)	0.126	0.16	(−0.13–0.44)
Men	95.23 (14.71)	91.19 (17.78)	0.103	0.25	(−0.12–0.63)
Women	84.60 (23.23)	85.55 (23.14)	0.706	−0.04	(−0.48–0.39)
Emotional health					
All	88.42 (27.00)	77.06 (39.09)	0.056	0.35	(0.06–0.64)
Men	92.54 (21.50)	78.03 (38.00)	0.019	0.51	(0.13–0.88)
Women	78.57 (37.30)	73.96 (41.25)	0.706	0.12	(−0.32–0.55)
Vitality					
All	66.65 (23.71)	65.00 (21.44)	0.448	0.07	(−0.21–0.36)
Men	72.30 (21.22)	66.48 (18.57)	0.075	0.29	(−0.09–0.66)
Women	55.27 (25.80	60.31 (23.07)	0.375	−0.20	(−0.64–0.23)
Mental health					
All	71.63 (21.93)	70.75 (20.54)	0.588	0.04	(−0.25–0.33)
Men	78.37 (18.01)	73.27 (19.07)	0.165	0.28	(−0.10–0.65)
Women	60.36 (23.94)	67.50 (21.62)	0.169	−0.31	(−0.74–0.13)
Physical component					
All	45.02 (8.66)	49.62 (7.11)	0.001	0.57	(0.28–0.86)
Men	46.31 (7.45)	51.14 (6.63)	0.001	0.67	(0.29–1.05)
Women	42.90 (10.45)	46.95 (6.74)	0.109	0.44	(0.00–0.87)
Mental component					
All	51.81 (9.92)	47.53 (12.51)	0.013	0.57	(0.28–0.86)
Men	54.31 (8.79)	48.08 (11.87)	0.001	0.62	(0.24–1.00)
Women	46.85 (11.94)	46.70 (12.87)	0.982	0.01	(−0.42–0.45)

Abbreviations: CI: confidence interval; FFS: functional fitness score; p50: percentile 50; SD: standard deviation.

**Table 6:** Association between physical and mental health vs. physical abilities measured by functional fitness battery tests.

	Physical Health					Mental Health				
	≤MeP Mean (SD)	>MeP Mean (SD)	*p*	Effect Size		≤MeP Mean (SD)	>MeP Mean (SD)	*p*	Effect Size	
				d	95% CI				d	95% CI
Functional fitness score										
All	3.39 (2.07)	4.32 (2.32)	0.003	0.42	(0.15–0.70)	4.04 (2.45)	3.62 (2.02)	0.199	0.19	(−0.08–0.46)
Men	3.46 (1.59)	4.25 (2.04)	0.028	0.44	(0.08–0.81)	4.31 (1.93)	3.41 (1.67)	0.004	0.51	(0.14–0.87)
Women	3.49 (1.62)	4.00 (1.81)	0.183	0.30	(−012–0.72)	3.68 (1.92)	3.79 (1.56)	0.737	−0.06	(−0.48–0.36)
Endurance										
All	62.86 (18.96)	72.68 (19.17)	0.001	0.52	(0.23–0.80)	68.45 (20.31)	66.67 (19.13)	0.498	0.09	(−0.19–0.37)
Men	66.47 (18.33)	77.69 (20.85)	0.009	0.58	(0.20–0.95)	74.04 (21.46)	64.69 (19.03)	0.216	0.23	(−0.14–0.60)
Women	56.95 (18.71)	66.30 (14.70)	0.023	0.56	(−0.11–1.00)	60.53 (15.66)	62.65 (18.77)	0.748	−0.12	(−0.56–0.32)
Lower body strength										
All	12.13 (3.18)	13.76 (3.71)	0.001	0.47	(0.20–0.75)	13.09 (3.50)	12.68 (3.53)	0.210	0.12	(−0.16–0.39)
Men	12.41 (3.09)	14.71 (4.48)	0.003	0.61	(0.24–0.98)	13.92 (3.78)	12.97 (3.95)	0.066	0.25	(−0.12–0.61)
Women	11.69 (3.32)	12.63 (2.04)	0.103	0.34	(0.08–0.76)	12.02 (2.81)	12.26 (2.80)	0.793	−0.08	(−0.50–0.34)
Sitting handgrip										
All	29.33 (11.04)	29.87 (11.08)	0.695	−0.05	(−0.32–0.22)	29.88 (11.83)	29.33 (10.40)	0.720	0.05	(−0.22–0.32)
Men	36.22 (8.03)	38.25 (6.40)	0.141	0.27	(0.09–0.64)	38.40 (7.90)	36.08 (6.94)	0.040	0.31	(−0.05–0.68)
Women	18.62 (4.65)	19.93 (6.06)	0.292	0.24	(0.18–0.66)	19.07 (5.31)	19.42 (5.53)	0.850	−0.06	(−0.48–0.35)
Standing handgrip										
All	29.33 (11.27)	29.57 (10.81)	0.757	−0.02	(−0.29–0.25)	29.74 (11.98)	29.20 (10.27)	0.679	0.05	(−0.22–0.32)
Men	36.02 (8.94)	37.65 (6.46)	0.250	−0.20	(−0.56–0.16)	38.33 (8.12)	35.48 (7.75)	0.019	0.36	(0.00–0.72)
Women	18.91 (4.60)	20.00 (6.02)	0.335	−0.20	(−0.62–0.22)	18.84 (5.34)	19.97 (5.34)	0.487	−0.21	(−0.63–0.21)
One-leg balance										
All	27.36 (20.20)	33.70 (19.62)	0.011	0.32	(0.04–0.59)	31.24 (20.40)	29.39 (19.98)	0.464	0.09	(−0.18–0.36)
Men	31.36 (20.36)	38.30 (18.69)	0.035	0.35	(0.01–0.72)	38.09 (19.16)	31.41 (20.09)	0.049	0.34	(−0.02–0.70)
Women	21.15 (18.48)	28.25 (19.51)	0.052	0.37	(0.05–0.79)	22.56 (18.72)	26.42 (19.66)	0.340	−0.20	(−0.62–0.22)
Agility										
All	6.38 (1.69)	5.90 (1.26)	0.052	0.32	(0.05–0.59)	6.26 (1.73)	6.09 (1.35)	0.862	0.11	(−0.16–0.39)
Men	6.00 (1.47)	5.59 (1.26)	0.148	0.29	(−0.07–0.66)	5.72 (1.42)	5.9 (1.37)	0.386	−0.13	(−0.49–0.23)
Women	6.98 (1.84)	6.27 (1.18)	0.090	0.46	(0.04–0.88)	6.94 (1.86)	6.36 (1.27)	0.233	0.37	(−0.05–0.79)
Upper body flexibility										
All	−14.34 (12.88)	−12.13 (11.96)	0.089	0.18	(0.10–0.45)	−13.07 (11.49)	−13.57 (13.28)	0.597	0.04	(−0.23–0.31)
Men	−16.44 (13.23)	−13.03 (13.64)	0.106	0.25	(0.11–0.62)	−13.17 (12.66)	−16.39 (13.96)	0.080	0.24	(−0.12–0.60)
Women	−11.06 (11.71)	−11.06 (9.65)	0.735	0.00	(−0.42–0.42)	−12.94 (9.97)	−9.43 (11.12)	0.081	−0.33	(−0.75–0.09)
Lower body flexibility										
All	−3.70 (9.18)	−1.10 (9.42)	0.060	0.28	(0.01–0.55)	−2.42 (9.51)	−2.62 (9.27)	0.561	0.02	(−0.25–0.29)
Men	−4.83 (9.47)	−1.37 (9.77)	0.079	0.36	(0.00–0.72)	−3.72 (9.68)	−3.11 (9.80)	0.984	−0.06	(−0.42–0.30)
Women	−1.94 (8.5)	−0.78 (9.10)	0.426	0.13	(0.29–0.55)	−0.77 (9.14)	−1.90 (8.49)	0.413	0.13	(−0.29–0.55)

Abbreviations: CI: confidence interval; MedP: Spanish population median.

## Data Availability

There are restrictions on the availability of data for this trial due to the signed consent agreements around data sharing, which only allow access to external researchers for studies following the project’s purposes. Requestors wishing to access the trial data used in this study can make a request to pep.tur@uib.es.
